# Prevalence and factors with potentially inappropriate prescribing among older outpatients with depression: a multicentre study across China

**DOI:** 10.7189/jogh.15.04216

**Published:** 2025-07-11

**Authors:** Fangyuan Tian, Zhaoyan Chen, Ying Zhang

**Affiliations:** 1 Department of Pharmacy, National Clinical Research Centre for Geriatrics, West China Hospital, Sichuan University, Chengdu, China; 2 Department of Epidemiology and Health Statistics, West China School of Public Health and West China Fourth Hospital, Sichuan University, Chengdu, China

## Abstract

**Background:**

Older outpatients with depression are at high risk for potentially inappropriate prescription (PIP). This investigation sought to determine the frequency and associated factors of PIP within Chinese older adults.

**Methods:**

This cross-sectional study used prescription data from older outpatients with depression from 90 hospitals in seven cities in China from January–December 2021. Risk factor identification for PIP employed multivariate logistic regression analysis. Trend assessment was performed through joinpoint regression to calculate the average annual percent change.

**Results:**

The prevalence of PIP reached 50.42%. The top five PIM were alprazolam, clonazepam, olanzapine, lorazepam, estazolam. The prevalence of PIP decreased from 51.56 to 50.99% (average annual percent change = −0.335%). Logistic regression demonstrated that tertiary-level hospital (odds ratio (OR) = 1.215; 95% confidence interval (CI) = 1.100, 1.342, *P* < 0.001), department of psychiatry (OR = 1.958; 95% CI = 1.855, 2.067, *P* < 0.001), age ≥80 (OR = 1.069; 95% CI = 1.016, 1.124, *P* = 0.01), more diseases (OR = 1.209; 95% CI = 1.092, 1.339, *P* < 0.001), polypharmacy (OR = 1.672; 95% CI = 1.541, 1.814, *P* < 0.001) exhibited positive links to PIP among older outpatients suffering from depression.

**Conclusions:**

This investigation revealed that the occurrence of PIP in older outpatients with depression is high in China.

Population aging has accelerated globally due to reduced birth rates and extended life spans [1]. Data from China's seventh national population census indicated that the proportion of individuals aged 65 and above expanded by 4.6% (from 8.9 to 13.5%) during the previous decade [2]. As the elderly demographic continues to expand, chronic disease prevalence has risen substantially, necessitating multiple medication usage among older adults. The escalating number of medications prescribed to older individuals, combined with alterations in pharmacokinetics and pharmacodynamics, elevates the likelihood of drug-drug interactions and adverse drug reactions under specific medical conditions.

The management of medicinal safety among older adults represents a crucial concern requiring broad consideration. Medications administered to older adults are deemed potentially inappropriate medications (PIM) when their possible adverse effects surpass their anticipated advantages [3]. The practice of including PIM in prescriptions constitutes potentially inappropriate prescribing (PIP) [4]. This emerges as a substantial element elevating medication safety hazard in older adults [5]. These instances stand as the third primary reason for hospitalisation and the predominant source of hospital-acquired conditions within the older population [6]. Research indicates that older adults receiving PIP demonstrate heightened risks regarding mortality, osteoporosis, falls, and subsequent hospital returns compared to those without such prescriptions, substantially diminishing their overall well-being [7].

For detecting and addressing PIP, multiple nations established PIM evaluation criteria [8]. The Beers criteria alongside Screening tool of older person’s prescriptions/screening tool to alert doctors to the right (STOPP/START) criteria represent prominent assessment tools in global practice. Regarding the Chinese context, researchers formulated domestic guidelines by synthesising international frameworks, analysing data obtained from three national adverse drug reactions surveillance centres, and examining reports collected from 22 Beijing-based hospitals. These guidelines underwent refinement through expert consensus utilising the Delphi methodology, leading to their formal publication as national criteria in 2018 [9].

The worldwide prevalence of PIP among older adults attending outpatient clinics is 36.7%, with a substantial increase in PIP frequency observed across this population over the past two decades [10]. Older adults with depression often receive antidepressants and sedative-hypnotic medications through outpatient services, which are commonly categorised as PIM; however, existing research has remained insufficient in examining the prevalence of PIP and its associated factors specifically among older Chinese outpatients diagnosed with depression. This study's findings are expected to elucidate the medication use patterns among older depressed outpatients, facilitate the development of targeted management strategies to enhance medication safety, and provide evidence-based guidance for improving health management practices in this vulnerable population.

## METHODS

### Study population, setting, and data source

The cross-sectional investigation obtained information from a prescription evaluation partnership initiative coordinated by the Chinese Pharmaceutical Association. This initiative examines prescription records, medication usage patterns, and pharmacoeconomic aspects within Chinese medical facilities. The investigation encompassed seven metropolitan areas followed established geographic frameworks. Through purposive selection, the investigation group identified multiple secondary and tertiary health care centres in each location. Regarding older patient prescriptions at these facilities, quarterly random sampling occurred across 10 days (divided into two five-day intervals). Medical centres equipped with hospital information systems transmit electronic prescription records through their platforms. For institutions lacking hospital information systems capabilities or containing incomplete electronic records, physical prescriptions underwent manual digital entry. Collected elements encompassed location identifiers, medical diagnoses, clinical divisions, medication details, product specifications, administration methods, dispensed amounts, dosage instructions, and patient demographics, encompassing sex and age. Any mention of depression in the record sufficient were depressive disorders defined. The analysis incorporated prescriptions for older (aged ≥65 years) individuals seeking outpatient depression treatment between 1 January–31 December 2021. The analysis excluded prescriptions containing solely non-medicinal components like injection water or imaging agents. This analysis followed the Strengthening the Reporting of Observational Studies in Epidemiology publication criteria [11].

### Diagnosis and medication classification

Medical prescriptions underwent classification and arrangement utilising the International Classification of Diseases, 10th Revision (ICD-10). Medications were categorised based on the World Health Organization's Anatomical Therapeutic Chemical classification system and generic drug names. A physician and pharmacist conducted prescription classification employing ICD-10 codes. The pharmacist allocated ICD-10 codes to diagnoses corresponding to the ICD-10 framework. For unmatched diagnoses, the physician applied professional knowledge to determine suitable codes. When coding difficulties arose, two physicians and one pharmacist collaborated to establish a consensus. For documentation lacking diagnostic information, the research group established telephone contact with respective health care facilities for verification. Two independent pharmacists executed medication categorisation. During instances of discordance, a senior pharmacist provided the definitive determination. Regarding prescriptions with incomplete medication information, the research group initiated telephone communication with respective health care facilities for verification.

### Evaluation criteria

In this study, Chinese criteria (2018 version) were utilised to evaluate PIM occurrence within outpatient prescriptions for older individuals experiencing depression. The assessment protocol involved independent evaluations by two clinical pharmacists with expertise in chronic conditions through an information platform. Subsequently, a geriatric clinical pharmacist performed manual verification of the findings. When initial evaluations showed differences, consensus was reached through collaborative discussions among the three specialists. The Chinese criteria consist of dual evaluation categories. Category one encompasses 72 criteria addressing 72 distinct medications, while category two contains 34 criteria covering 44 medications linked to 27 specific medical conditions. The complete framework includes 106 evaluation criteria. The organisation of medications follows pharmacological actions for the 72 items in category one, and disease-state associations for the 44 medications connected to 27 conditions. A prescription qualifies as PIP when containing any medication from either the 72-item list or the 44 medications associated with the 27 medical conditions specified in Chinese criteria. Each PIP is assessed on the basis of the number of PIM entries it contains according to the aforementioned criteria. If a prescription contains one of the 106 PIMs, it is defined as a single PIM-related PIP. If it contains two or more of the 106 PIMs, it is defined as multiple PIM-related PIPs.

### Statistical analysis

Frequency measurements characterised categorical information, while group comparisons utilised χ^2^ analysis for categorical variables. For normally distributed continuous variables, data representation employed mean ± standard deviation (SD), whereas non-normally distributed continuous variables utilised median ± interquartile range (IQR). Multivariate logistic regression analysis examined association between risk factors and PIM utilisation (non-PIP = 0, PIP = 1) to assess factor impacts on PIP. The regression outcomes included odds ratios (ORs) and 95% confidence intervals (CIs). Statistical computations utilised the *R* software platform, version 4.2.0 (*R* Core Team, Vienna, Austria). Trend alterations underwent evaluation through average annual percentage change employing a Poisson regression model [12]. The analyses and plots were generated via the software Joinpoint Regression Program, 5.0.2, (US National Cancer Institute Bethesda, Maryland, USA). Statistical significance was established at a two-sided *P* < 0.05.

### Ethical approval

Approval was provided by the West China Hospital Research Ethics Board (2024/810). No individual patient consent was needed, as all the data were deidentified.

## RESULTS

### Prescription characteristics

**Figure 1:**
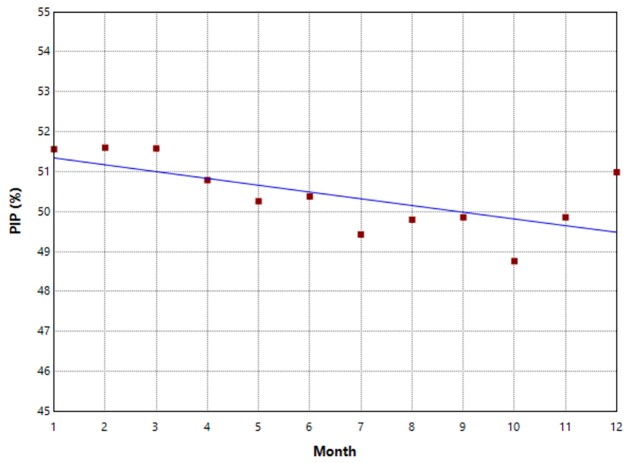
Trends of PIP in older outpatients with depression. PIP – potentially inappropriate prescribing.

A sum of 90 hospitals, including 40 516 outpatient prescriptions, were analysed for older adults with depression; the prevalence of PIP was calculated by year, which was 50.42%. Among the seven cities, the prevalence of PIP in Guangzhou was the highest (67.64%), whereas the prevalence of PIP in Beijing was the lowest (42.38%). Secondary-level hospitals accounted for 844 PIP (40.44%), whereas tertiary-level hospitals accounted for the majority, with 19 584 PIP (50.96%). The prevalence of the department of psychiatry was the highest (57.70%), whereas the prevalence of the department of geriatrics was the lowest (36.09%). Male patients received 13 787 PIP (50.43%), whereas female patients received 6641 PIP (50.39%). The average age stood at 72 (IQR = 68, 79) years, spanning from 65 to 103 years, where older patients (≥80 years of age) comprised 49.57% of prescription recipients. The PIP occurrence peaked in the initial quarter (51.58%) of the four quarters. The typical disease count was two (IQR = 1, 3), and 51.05% of individuals exhibited multiple chronic conditions alongside depression. Concerning prescription medications, the typical count remained at two (IQR = 1, 3), with 10.22% of outpatients (n = 4139) exhibiting polypharmacy. The PIP rates appeared elevated in free payment categories *vs*. other payment methods (**Table 1**). The analysis revealed that 37.19% (n = 15 068) of depression patients exhibited anxiety, 23.84% (n = 9660) experienced sleep disorder, and 3.91% (n = 1585) presented with dementia (**Table 2**). Throughout January–December, PIP prevalence among older outpatients with depression in China demonstrated a modest reduction, declining from 51.56 to 50.99%, indicating a notable descending pattern (average annual percentage change: −0.335%; 95% CI = −0.598, −0.071) (**Figure 1**).

**Table 1:** Basic characteristics of older outpatients with depression

Characteristics	Total (n)	PIP (n)	PIP (%)	χ^2^	*P*-value
**Total**	40 516	20 428	50.42		
**City**				945.174	<0.001
Beijing	10 950	4641	42.38		
Chengdu	4629	2112	45.63		
Guangzhou	4162	2815	67.64		
Hangzhou	3009	1598	53.11		
Shanghai	13 824	6967	50.40		
Tianjin	2322	1280	55.12		
Zhengzhou	1620	1015	62.65		
**Hospital level**				87.646	<0.001
2nd	2087	844	40.44		
3nd	38 429	19 584	50.96		
**Department**				944.109	<0.001
Psychiatry	20 951	12 089	57.70		
Geriatrics	1305	471	36.09		
Other	18 260	7868	43.09		
**Sex**				0.005	0.944
Male	27 338	13 787	50.43		
Female	13 178	6641	50.39		
**Age group, years (IQR)**	72 (68, 79)			3.533	0.06
65–79	31 189	15 805	50.67		
≥80	9327	4623	49.57		
**Quarter of visit**				8.68	0.034
Q1	10 291	5308	51.58		
Q2	10 408	5252	50.46		
Q3	9282	4614	49.71		
Q4	10 535	5254	49.87		
**No. of diseases (IQR)**	2 (1, 3)			136.67	<0.001
1	19 831	10 294	51.91		
2–4	14 288	7336	51.34		
≥5	6397	2798	43.74		
**No. of medications (IQR)**	2 (1, 3)			14.022	<0.001
1–4	36 377	18 227	50.11		
≥5	4139	2201	53.18		
**Payment**				237.556	<0.001
Free	12 779	6932	54.25		
Partial fee	17 879	8248	46.13		
Full fee	9858	5248	53.24		

IQR – interquartile range, PIP – potentially inappropriate prescribing

**Table 2:** The prevalence of PIP in older outpatients with depression combined with other disease

Characteristics	Total	PIP (n)	PIP (%)	χ^2^	*P*-value
Total	40 516	20 428		50.42	
Anxiety					
*Yes*	15 068	7422	49.26	12.978	<0.001
*No*	25 448	13 006	51.11		
Sleep disorder					
*Yes*	9660	5827	60.32	497.441	<0.001
*No*	30 856	14 601	47.32		
Dementia					
*Yes*	1585	614	38.74	90.041	<0.001
*No*	38 931	19 814	50.90		
Hypertension					
*Yes*	4820	1886	39.13	279.001	<0.001
*No*	35 696	18 542	51.94		
Coronary heart disease					
*Yes*	2751	1074	39.04	152.878	<0.001
*No*	37 765	19 354	51.25		
Diabetes					
*Yes*	1940	682	35.15	279.001	<0.001
*No*	38 576	19 746	51.19		
Arthritis					
*Yes*	306	111	36.27	24.678	<0.001
*No*	40 210	20 317	50.53		
Hyperlipidaemia					
*Yes*	2806	1081	38.52	170.638	<0.001
*No*	37 710	19 347	51.30		
Osteoporosis					
*Yes*	949	352	37.09	69.052	<0.001
*No*	39 567	20 076	50.74		
Cerebrovascular disease					
*Yes*	1957	880	44.97	24.458	<0.001
*No*	38 559	19 548	50.70		

PIP – potentially inappropriate prescribing

### PIP and PIM

Among the 20 428 PIPs, 15 556 (76.15%) were identified with at least one PIM, and 4972 were identified as multiple PIM-related PIPs. The 20 428 PIPs encompassed 26 113 PIMs, and the top five medications determined as inappropriate per the specified criteria consisted of clopidogrel, followed by alprazolam, clonazepam, olanzapine, lorazepam, estazolam. These five medications accounted for 65.42% of all PIMs (Table S1 in the **Online Supplementary Document**).

### Factors for PIP

The logistic regression analysis revealed that treatment at tertiary-level hospitals (OR = 1.215; 95% CI = 1.100, 1.342, *P* < 0.001), visits to psychiatric departments (OR = 1.958; 95% CI = 1.855, 2.067, *P* < 0.001), age ≥80 (OR = 1.069; 95% CI = 1.016, 1.124, *P* = 0.01), multiple comorbidities (OR = 1.209; 95% CI = 1.092, 1.339, *P* < 0.001), and polypharmacy (OR = 1.672; 95% CI = 1.541, 1.814, *P* < 0.001) exhibited positive association with PIP among older outpatients experiencing depression. The analysis indicated that department of geriatrics (OR = 0.779; 95% CI = 0.686, 0.886, *P* < 0.001), partial fee (OR = 0.735; 95% CI = 0.690, 0.783, *P* < 0.001), and full fee (OR = 0.783; 95% CI = 0.730, 0.841, *P* < 0.001) demonstrated negative associations with PIP (**Table 3**).

**Table 3:** Multivariate logistic regression analysis of factors associated with PIP in older outpatients with depression

Characteristics	OR	95% CI	*P*-value
**City**			
Beijing	Reference		
Chengdu	1.439	1.328, 1.559	<0.001
Guangzhou	3.543	3.238, 3.876	<0.001
Hangzhou	1.968	1.783, 2.171	<0.001
Shanghai	1.409	1.294, 1.533	<0.001
Tianjin	1.913	1.724, 2.122	<0.001
Zhengzhou	3.393	3.016, 3.817	<0.001
**Hospital level**			
2nd	Reference		
3nd	1.215	1.100, 1.342	<0.001
**Department**			
Other	Reference		
Psychiatry	1.958	1.855, 2.067	<0.001
Geriatrics	0.779	0.686, 0.886	<0.001
**Sex**			
Female	Reference		
Male	1.041	0.996, 1.088	0.075
**Age group, years**			
65–79	Reference		
≥80	1.069	1.016, 1.124	0.010
**Quarter of visit**			
Q1	Reference		
Q2	0.960	0.907, 1.017	0.164
Q3	0.929	0.876, 0.985	0.014
Q4	0.937	0.885, 0.991	0.024
**No. of diseases**			
1	Reference		
2–4	1.147	1.076, 1.223	<0.001
≥5	1.209	1.092, 1.339	<0.001
**No. of medications**			
1–4	Reference		
≥5	1.672	1.541, 1.814	<0.001
**Payment**			
Free	Reference		
Partial fee	0.735	0.690, 0.783	<0.001
Full fee	0.783	0.730, 0.841	<0.001
**Disease**			
Anxiety	1.045	0.996, 1.097	0.071
Sleep disorder	2.808	2.642, 2.984	<0.001
Dementia	0.857	0.765, 0.959	0.007
Hypertension	0.685	0.631, 0.743	<0.001
Coronary heart disease	0.791	0.716, 0.874	<0.001
Diabetes	0.597	0.534, 0.667	<0.001
Arthritis	0.804	0.620, 1.043	0.100
Hyperlipidaemia	0.989	0.895, 1.092	0.820
Osteoporosis	0.786	0.675, 0.916	0.002
Cerebrovascular disease	1.033	0.931, 1.145	0.544

CI – confidence interval, OR – odds ratio

## DISCUSSION

This investigation evaluated 40 516 prescriptions given to older individuals diagnosed with depression across 90 medical facilities in seven Chinese cities, utilising the 2018 Chinese PIM guidelines. The analysis identified 20 428 instances of PIP, indicating a PIP occurrence rate of 50.42%. This percentage exceeds the reported rates among older individuals diagnosed with Alzheimer disease (39.43%) [13] and those with cancer (34.37%) [14]. Patients with Alzheimer often suffer from psychiatric symptoms in addition to their primary disease. In these cases, both the patients and their families tend to favour the use of antipsychotic and sedative-hypnotic medications to control behavioural symptoms, which are frequently classified as PIM [15,16]. Similarly, older cancer patients may use antidepressants and sedative-hypnotics due to emotional distress, further contributing to the use of PIM. However, the demand for these medications is lower in these populations compared to elderly patients with depression, which may explain the differences in the prevalence of PIP across patient groups. The relatively high prevalence of PIP among older patients with depression in China suggests that long-term medication use is common in this population, raising significant concerns about drug safety. Given the serious risks associated with PIM, we recommend that governmental agencies implement policies to regulate PIM and PIP and that health insurance authorities introduce reimbursement controls for these medications.

To further improve the management of PIP among older adults and ensure rational drug use, China released guidelines for PIM in 2018. The application of these guidelines in outpatient services should be further strengthened, and awareness of PIP among health care professionals and older adults should be increased. Research examining 15 932 older patients in Canada between 2011–2019 investigated how discharge medication review interventions affected PIP prevalence. Following the 2015 intervention implementation, data showed declining PIP rates among older inpatients discharged from tertiary medical centres [17]. An interrupted time-series analysis study analysed 50 924 prescriptions across eight older outpatient facilities in the USA during 2000–2004, examining computerised decision support systems' influence on PIP. The findings demonstrated no apparent effect of PIP occurrence after system deployment (level change (*P* = 0.52) or slope change (*P* = 0.27)) [18]. The effects of a guideline issued by the National Health Commission of China in 2018 on the use of carbapenems in Shaanxi Province between 2017–2020 were analysed via interrupt time series. The results revealed that the use frequency of carbapenems tended to decrease after the release of the guideline [19]. Additionally, an interrupted time series investigation examined how National Health Commission guidelines influenced key monitored medication usage at a Xi'an tertiary hospital from 2014 to 2021. The analysis revealed a significant decline in both utilisation and expenses of monitored medications post-guideline implementation, with the analysis showing that total monthly defined daily does (DDDs) decreased by 430 (*P* < 0.001) and spending reduced by 4 682 USD (*P* = 0.003) in the 19-month post-period compared to the pre-policy trend [20]. These results collectively indicate that effective interventions or guidelines can significantly improve rational drug use in hospitals.

The prevalence of PIP in Beijing is lower compared to other cities (*P* < 0.001). This may be related to the fact that the Chinese criteria for PIM were primarily developed by medical institutions in Beijing. Consequently, local clinicians are more familiar with the risks of PIP in older patients and are more attentive to PIM issues when prescribing. The prevalence of PIP in secondary-level hospitals is higher than in tertiary-level hospitals. This could be due to the fact that patients at secondary-level hospitals have less severe conditions and lower comorbidity rates than those at tertiary-level hospitals, which may contribute to a lower prevalence of PIP. Patients attending department of geriatric have a lower risk of PIP, likely because geriatric specialists are more knowledgeable about the Chinese criteria and are more vigilant in managing PIMs. These findings indicate potential correlations rather than robust causal relationships, acknowledging that the observed effects may be influenced by unmeasured confounders.

The frequency of PIP escalated in association with medication quantity. A meta-analysis indicates that polypharmacy functions as a standalone risk element for PIP [21], consistent with these observations. The probability of PIP exposure rises by approximately 5.2% per additional medication incorporated in the PIM criteria [22]. The analysis demonstrates that patients with multiple comorbidities were more likely to receive PIP, potentially attributed to expanded diagnostic classifications and prescribed drug quantities, elevating PIP likelihood. Medications qualifying for complete reimbursement demonstrate the highest PIP occurrence, whereas non-reimbursed prescriptions show minimal rates. This pattern possibly stems from reimbursement structures incentivising increased medication prescriptions, subsequently amplifying PIP risk.

In the current study, while it was identified that certain prescriptions included multiple PIMs, a deeper exploration of the implications is warranted. The co-prescription of multiple PIMs may lead to a cumulative risk, potentially increasing the likelihood of adverse drug events, drug-drug interactions, and worsening health outcomes. Additionally, there may be specific patterns in co-prescribing, such as combinations of sedative-hypnotics, that require special attention as they could pose heightened risks to patients. Future research should focus on quantifying this cumulative risk and identifying common co-prescribing patterns to inform targeted interventions aimed at improving medication safety and rational prescribing practices.

The top five detected drugs included four benzodiazepines. The extensive utilisation of benzodiazepine medications correlates with the substantial occurrence of sleep disorders in older adults. Although approximately 80% of individuals receive prescriptions for sleep-related difficulties, the majority of prescribed benzodiazepines and sleep-inducing agents prove inappropriate for older adults [23]. As physiological aging progresses in organs and tissues, older adults exhibit heightened medication sensitivity. Although benzodiazepines demonstrate links to elevated risks of cognitive decline, falling incidents, and bone fractures, physicians continue prescribing them frequently to older adults [24]. Current medical protocols suggest initiating insomnia management in older adults through non-medicinal approaches, encompassing environmental modification, sleep scheduling, tension reduction exercises, and psychological interventions [25]. A review reported that cognitive behavioural therapy is considered the first-line treatment for insomnia, with benzodiazepines being discouraged for older adults, especially for long-term use [26]. Z-category medications emerge as the preferred pharmaceutical option for insomnia treatment, considering their metabolic characteristics and safety parameters. Nevertheless, scientific investigations indicate these compounds present comparable adverse reaction risks to benzodiazepines, including balance disruption and elevated fall probability in older adults [27]. An analytical comparison between zolpidem and benzodiazepines demonstrated that zolpidem administration correlates with increased fracture likelihood compared to alprazolam and lorazepam [28].

Implementing pharmacist-led review systems in outpatient psychiatric clinics could mitigate risks by identifying benzodiazepine overuse and polypharmacy-related interactions. Revising insurance reimbursement policies to incentivise evidence-based prescribing for high-risk medications (*e.g.* benzodiazepines) may curb inappropriate use. Developing clinical guidelines and provider training focused on benzodiazepine stewardship and polypharmacy risk assessment for psychiatric care settings.

This investigation presents certain constraints that warrant consideration. First, despite collecting data from multiple medical centres across seven cities throughout China, the study scope encompassed only older outpatients at secondary and tertiary medical facilities. Consequently, the significance of these findings regarding PIP prevalence among older individuals with depression in primary care environments remains unexplored. Second, the absence of follow-up information for older outpatients prevented a comprehensive evaluation of adverse health consequences resulting from PIP. Additionally, the investigation could not determine the influence of the Chinese criteria on specific clinical outcomes, including adverse drug event occurrence or overall mortality rates. Third, Stratified analysis was not performed in this study. This represents a methodological limitation, as failure to control variables like comorbidity burden may have obscured subgroup-specific PIP trends. Future research is recommended to incorporate stratified designs to enhance result interpretability.

## CONCLUSIONS

Clinicians should prioritise evidence-based guidelines, reducing benzodiazepine use and implementing non-pharmacological therapies for older adults with depression, while adopting pharmacist-led interdisciplinary medication reviews to address polypharmacy risks (OR = 1.672) and train on the Chinese criteria, especially in tertiary hospitals (OR = 1.215) and psychiatric departments (OR = 1.958). Policy-wise, revise insurance reimbursement to disincentivise PIM prescribing and expand national stewardship programmes like the Key Monitoring Drugs initiative, targeting cities with high PIP prevalence. Future research should validate findings in primary care, analyse long-term outcomes of PIP on adverse events, and test intervention efficacy to inform evidence-based practices and improve medication safety for this vulnerable population.

## Additional material


Online Supplementary Document
