# Supplementary material for: Prevalence and factors with potentially inappropriate prescribing among older outpatients with depression: a multicentre study across China
Source: J Glob Health. 2025 Jul 11;15:04216. doi: 10.7189/jogh.15.04216 (PMC12256811; doi:10.7189/jogh.15.04216)
Supplement: Online Supplementary Document [file jogh-15-04216-s001.pdf]

Supplement to: Fangyuan Tian, Zhaoyan Chen, Ying Zhang. Prevalence and factors with potentially inappropriate prescribing among older outpatients with depression: a multicentre study across China. J Glob Health. 2025;15:04216.

Table S1. PIM used in the prescriptions

Characteristic	Beijing (n = 10 950)	Chengdu (n = 4629)	Guangzhou (n = 4162)	Hangzhou (n = 3009)	Shanghai (n = 13 824)	Tianjin (n = 2322)	Zhengzhou (n = 1620)	Total (n = 40 516)
PIP	4641	2112	2815	1598	6967	1280	1015	20 428
Single PIM-related PIP	3767	1707	1812	1172	5266	954	878	15 556
Multiple PIM-related PIP	874	405	1003	426	1701	326	137	4872
PIM	5674	2566	3998	2122	8925	1651	1177	26 113
Alprazolam	204	653	1053	304	1001	124	448	3787
Clonazepam	582	432	476	233	1665	129	140	3657
Olanzapine	687	454	602	351	855	308	54	3311
Lorazepam	1160	155	454	148	882	394	10	3203
Estazolam	904	338	192	185	813	315	377	3124
Total	3537	2032	2777	1221	5216	1270	1029	17 082

PIM – potentially inappropriate medication, PIP – potentially inappropriate prescribing
